# Paper for Water-Closets

**Published:** 1876-07

**Authors:** 


### PAPER FOR WATER-CLOSETS.

Many of the ills that burden life come from want of attention to matters that are very generally regarded as trivial. How few stop to consider that the use of newspapers, pamphlets, old books, and in fact all sorts of printing paper, in the water-closet, is an evil more pernicious in its results than the greatest pestilence that ever scourged the land. When it is considered that printing paper contains chloride of lime, and that printing ink contains turpentine, our views will not seem extravagant. The great prevalence of piles, even among children, induced by the use of printing paper in water-closets, is alarming. We can use no milder word in view of the many and terrible diseases that have their beginning in *mild* attacks of this almost general ailment.

More than half the adult population of this country suffer more or less from constipation of the bowels. There is hardly a case that has not been preceded by piles, and hardly a case of piles that has not been brought on by the use of printing paper. These troubles do not rank among the minor ills of life, as many suppose. They *always* precede those dreaded cancerous affections of the parts adjacent. They either induce or aggravate diseases of the bladder, prostate, and kidneys. And these are not all the evils that follow and proceed from constipation. When effete matters do not have free exit from the body, and are retained in the system an hour too long, they commence their destructive work. The blood is poisoned, the functions of the liver are deranged, and every vital organ is more or less affected.

It would be impossible to form an estimate of the proportion of premature deaths from a multitude of diseases whose origin could be traced to want of attention to the condition of the bowels. Almost unlimited opportunities for tracing the origin of many fatal diseases convince us that there is nothing so essential to health, and, consequently, to happiness, as careful attention to the demands of nature in this respect. The same watchful care should be extended to our children, if we would ensure to them sound health and long life, in comparison to which riches are as dross.

We have said that the habitual use of printing paper for the water-closet was the beginning of a train of evils, the end of which no man could prophecy. The reason why will suggest itself to most readers. But we wish every one who reads this article to comprehend it, and we will therefore explain our meaning more fully.

Any *irritating* substance applied to a healthy tissue will produce *irritation.* Repeated *irritation* produces *inflammation*, and an *inflamed* organ can not properly perform its function.

If *inflammation* is induced by repeated applications of chloride of lime, and other irritants found in printing paper, it impairs the healthy action of the bowels, and there results constipation and piles.

Until within a comparatively recent period, the remedy for these ills has been purgatives or injections. But the evils resulting from the treatment were only second to the diseases themselves. It is certainly not very creditable to the skill of the medical profession that they should have practiced this destructive treatment for ages, and that the precise location of the trouble, and its remedy, should have been discovered by Nelaton within the past twenty years. He was the greatest surgeon that has ever lived in any age or in any country. If he profited by his knowledge, it was his right to do so. We have prescribed several different kinds of suppositories, some of which we have found to answer well for piles only. But as piles depend upon constipation of the bowels, it is important to remove the cause of the trouble as promptly as possible. When the cause is removed, the trouble ceases.

As a substitute for purgatives, and a cure for piles, there is nothing, in our opinion, equal to "Nelaton's Suppository." It acts promptly, relieving the patient in an hour or two even where the inflammation is severe.

We have known many cases of habitual constipation of years' standing completely cured by "Nelaton's Suppository," so that the use of all remedies could be dispensed with afterward.

